# Identification of Heterogeneous Subtypes of Mild Cognitive Impairment Using Cluster Analyses Based on PET Imaging of Tau and Astrogliosis

**DOI:** 10.3389/fnagi.2020.615467

**Published:** 2021-01-26

**Authors:** Hyun Jeong Lee, Eun-Chong Lee, Seongho Seo, Kwang-Pil Ko, Jae Myeong Kang, Woo-Ram Kim, Ha-Eun Seo, Sang-Yoon Lee, Yeong-Bae Lee, Kee Hyung Park, Byeong Kil Yeon, Nobuyuki Okamura, Duk L. Na, Joon-Kyung Seong, Young Noh

**Affiliations:** ^1^Gil Medical Center, Gachon University College of Medicine, Incheon, South Korea; ^2^School of Biomedical Engineering, Korea University, Seoul, South Korea; ^3^Department of Neuroscience, College of Medicine, Gachon University, Incheon, South Korea; ^4^Department of Preventive Medicine, Gachon University College of Medicine, Incheon, South Korea; ^5^Department of Psychiatry, Gil Medical Center, Gachon University College of Medicine, Incheon, South Korea; ^6^Neuroscience Research Institute, Gachon University, Incheon, South Korea; ^7^Department of Neurology, Gil Medical Center, Gachon University College of Medicine, Incheon, South Korea; ^8^Division of Pharmacology, Faculty of Medicine, Tohoku Medical and Pharmaceutical University, Sendai, Japan; ^9^Department of Neurology, Samsung Medical Center, Sungkyunkwan University School of Medicine, Seoul, South Korea; ^10^Neuroscience Center, Samsung Medical Center, Seoul, South Korea; ^11^Department of Artificial Intelligence, Korea University, Seoul, South Korea; ^12^Interdisciplinary Program in Precision Public Health, Korea University, Seoul, South Korea; ^13^Department of Health Science and Technology, GAIHST, Gachon University, Incheon, South Korea

**Keywords:** mild cognitive impairment, tau, THK, cluster analysis, positron emission tomography

## Abstract

**Background:** Mild cognitive impairment (MCI) is a condition with diverse causes and clinical outcomes that can be categorized into subtypes. [^18^F]THK5351 has been known to detect reactive astrogliosis as well as tau which is accompanied by neurodegenerative changes. Here, we identified heterogeneous groups of MCI patients using THK retention patterns and a graph theory approach, allowing for the comparison of risk of progression to dementia in these MCI subgroups.

**Methods:** Ninety-seven participants including 60 MCI patients and individuals with normal cognition (NC, *n* = 37) were included and undertook 3T MRI, [^18^F]THK5351 PET, and detailed neuropsychological tests. [^18^F]Flutemetamol PET was also performed in 62 participants. We calculated similarities between MCI patients using their regional standardized uptake value ratio of THK retention in 75 ROIs, and clustered subjects with similar retention patterns using the Louvain method based on the modularity of the graph. The clusters of patients identified were compared with an age-matched control group using a general linear model. Dementia conversion was evaluated after a median follow-up duration of 34.6 months.

**Results:** MCI patients were categorized into four groups according to their THK retention patterns: (1) limbic type; (2) diffuse type; (3) sparse type; and (4) AD type (retention pattern as in AD). Subjects of the limbic type were characterized by older age, small hippocampal volumes, and reduced verbal memory and frontal/executive functions. Patients of the diffuse type had relatively large vascular burden, reduced memory capacity and some frontal/executive functions. Co-morbidity and mortality were more frequent in this subgroup. Subjects of the sparse type were younger and declined only in terms of visual memory and attention. No individuals in this subgroup converted to dementia. Patients in the AD type group exhibited the poorest cognitive function. They also had the smallest hippocampal volumes and the highest risk of progression to dementia (90.9%).

**Conclusion:** Using cluster analyses with [^18^F]THK5351 retention patterns, it is possible to identify clinically-distinct subgroups of MCI patients and those at greater risk of progression to dementia.

## Introduction

Mild cognitive impairment (MCI) is a pathologically and clinically heterogeneous disease characterized by lower cognitive performance, and is considered to be a transitional state between normal cognition and dementia (Petersen et al., 1999; Brooks and Loewenstein, 2010). Earlier efforts by our group have sought to identify characteristics of MCI to better predict clinical outcomes (Cummings et al., 2007). Previous studies have reported that amnestic MCI (aMCI) patients are at higher risk of progressing to Alzheimer's dementia (AD) (Guillozet et al., 2003; Grundman et al., 2004), while development in patients with non-amnestic MCI (naMCI) has been characterized by more diverse features, including frontotemporal dementia (FTD), dementia with Lewy bodies (DLB), small vessel disease, geriatric depression, and AD (Schneider et al., 2009; Ferman et al., 2013; Dugger et al., 2015). Histologically, patients with aMCI tend to have increased neurofibrillary tangles (NFTs), a hallmark feature of AD together with amyloid plaques (Dugger et al., 2015). In addition, patients with negative amyloid pathology based on amyloid positron emission tomography (PET) findings have been found to be less likely to progress to AD (Landau et al., 2016).

NFT pathology can now be visualized *in vivo* thanks to the development of radiotracers for tau imaging (Xia et al., 2013; Villemagne et al., 2014; Okamura et al., 2016). Tau PET imaging data closely correlates with cerebral atrophy and cognitive disorders, and has been very consistent with autopsy findings (Villemagne et al., 2014; Cho et al., 2016). Ongoing PET studies using tau-targeted tracers are expected to provide greater insight into the pathology associated with MCI. However, there have been recent concerns raised in regards to off-target tracer binding to monoamine oxidase-B (MAO-B) in the whole brain (Harada et al., 2018). Evidence suggests that [^18^F]THK5351 binding to MAO-B markedly contributes to *in vivo* tau PET signal. Recent studies showed [^18^F]THK5351 may be a suitable imaging marker to detect neurodegenerative changes caused by reactive astrogliosis as well as tau (Brendel et al., 2017; Ishiki et al., 2017; Schönecker et al., 2019). Several reports on tau distribution in MCI subtypes have been published in recent years (Okamura et al., 2015), with tau PET studies using [^18^F]THK5317 and [^18^F]AV-1451 showing that tau PET tracers can help distinguish patients with mild cognitive impairment from normal controls, with the topography of tau PET retention including [^18^F]THK5351 in MCI patients being generally similar across the tau PET tracers used (Chiotis et al., 2016; Cho et al., 2016; Johnson et al., 2016). However, to our knowledge, there have not been any previous efforts to classify subgroups of MCI patients according to astrogliosis and tau deposition patterns.

MCI is a heterogeneous disease entity that may be caused by various etiologies and thus has a different prognosis. There is no established biomarker to confirm progression yet, and APOE or amyloid PET may tell whether to convert to AD. However, in clinical view, all amyloid negatives patients do not have stable prognosis, and in some cases, progression to dementia in relation to vascular factors, other neurodegenerative pathologies or even comorbidity may occur. As mentioned above, it is known that THK5351 PET does not bind to AD type tau pathology specifically due to MAO-B availability, but on the other hand, we presumed if it could show pathologic changes related to neurodegeneration more sensitively.

The objectives of this study were to investigate variable topographical patterns of neurodegenerative changes in MCI patients using [^18^F]THK5351 which detect combined reactive astrogliosis and tau, and determine whether MCI patients can be categorized into distinct subtypes according to THK retention patterns using cluster analyses. Furthermore, we sought to identify clinical features and the risk of conversion to dementia for each MCI subtype.

## Materials and Methods

### Participants

Sixty participants including MCI patients (*n* = 60) who met Petersen's criteria: (i) self- or informant-reported cognitive complaint, (ii) objective cognitive impairment for age, (iii) preserved independence in functional abilities, and (iv) no dementia (Petersen et al., 2014), as well as participants with normal cognition (NC, *n* = 37) were recruited. All participants underwent [^18^F]THK5351 PET scans, 3.0-Tesla MRI scans, and detailed neuropsychological tests at Gachon University Gil Medical Center. Sixty two participants out of 97 took [^18^F]flutemetamol PET scans. All subjects also completed clinical interviews for medical history and underwent neurologic examinations, including the Mini-Mental State Examination (MMSE) and Clinical Dementia Rating-Sum of Boxes (CDR-SOB), and detailed neuropsychological tests described in Supplementary Material 1.

We excluded participants with high-signal abnormalities on MRI scan, such as intracranial hemorrhage, traumatic brain injury, leukodystrophy, multiple sclerosis, or vasculitis. Laboratory tests were conducted to rule out secondary causes of cognitive decline, and included complete blood counts, vitamin B12, folate levels, a metabolite profile, thyroid function tests, and syphilis serology. APOE genotyping was also conducted for all participants. Although we could not perform genetic tests for familial AD with autosomal inheritance, such as PSEN 1, PSEN 2, and APP genes, none of the patients had a family history suspicious for autosomal dominant AD; ≥2 first degree relatives with a history of dementia or ≥1 family member presenting with dementia at an extremely young age.

Subjects, who scored below −1.0 SD of the norm in at least one memory test (Seoul Verbal Learning Test, delayed recall [SVLT-DR] or Rey Complex figure test, delayed recall [RCFT-DR]), were placed into the aMCI group. Participants who scored below −1.0 SD of the norm in at least one test in the cognitive domains (language, visuospatial and frontal/executive functions) other than memory were placed in the naMCI group (Petersen, 2004).

Thirty-seven NC participants with no history of neurological or psychiatric illness and no abnormalities detected on neurologic examination were included. In the NC group, CDR-SOB scores were zero and neuropsychological test performance was defined as above −1.0 SD. All NC subjects were amyloid negative on [^18^F]flutemetamol PET scans.

We obtained written informed consent from each participant, and the Institutional Review Board of Gachon University Gil Medical Center approved the study.

### Image Acquisition and Pre-processing

#### MR Imaging Acquisition and Segmentation

All images were acquired with a 3.0T MRI (Verio, Siemens with a Siemens matrix coil). The 3D T1-Magnetization-Prepared Rapid Gradient-Echo (MPRAGE) imaging parameters used were as follows: repetition time = 1,900 ms, echo time = 2.93 ms, flip angle = 8°, pixel bandwidth = 170 Hz/pixel, matrix size = 256 × 208, field of view = 256 mm, NEX = 1, total acquisition time = 4 min 10 s, voxel size = 1.0 × 1.0 × 1.0 mm^3^.

Other clinical MRI sequences including the fluid attenuated inversion recovery (FLAIR), susceptibility weighted imaging (SWI) and T1- and T2-weighted imaging were also acquired. The FLAIR imaging parameters used were as follows: repetition time = 9,000 ms, echo time = 122 ms, flip angle = 150°, pixel bandwidth = 287 Hz/pixel, matrix size = 256 × 224. The SWI imaging parameters used were as follows: repetition time = 27 ms, echo time = 20 ms, flip angle = 15°, pixel bandwidth = 120 Hz/pixel, matrix size = 256 × 224. T1-weighted imaging parameters used were as follows: repetition time = 500 ms, echo time = 9.2 ms, flip angle = 70°, pixel bandwidth = 391 Hz/pixel, matrix size = 256 × 224. T2-weighted imaging parameters used were as follows: repetition time = 9,650 ms, echo time = 88 ms, flip angle = 120°, pixel bandwidth = 174 Hz/pixel, matrix size = 256 × 224.

Lacunes were defined as lesions of ≥3 and ≤ 15 mm in diameter with low signal on T1-weighted images, high signal on T2-weighted images, and a perilesional halo on 80 axial sections of FLAIR images. Microbleeds were defined as small lesions of ≤ 10 mm in diameter, using criteria proposed by Greenberg et al. (2009), on 20 axial sections of time constant for T2-weighted gradient–recalled echo sequence MRIs. Details of the measurement methods for the lacunes and microbleeds are presented in Supplementary Material 2.

Images were analyzed using FreeSurfer 6.0 (www.surfer.nmr.mgh.harvard.edu), and MRI parcellation was performed as described previously (Kang et al., 2017).

#### PET Imaging Acquisition

All PET scans were acquired with a Siemens Biograph 6 Truepoint PET/computed tomography (CT) scanner (Siemens, Knoxville, Tennessee, USA) using a list-mode emission acquisition. [^18^F]THK5351 was synthesized and radiolabeled at Gachon University Neuroscience Research Institute. THK PET involves a 20-min emission scan starting 50 min after 185 MBq of [^18^F]THK5351 is injected intravenously (50–70 min). β-amyloid imaging was obtained using [^18^F]-flutemetamol (FLUTE) PET scans. Among the 97 participants, 62 participants underwent a 20-min emission scan starting 90 min after the intravenous injection of 185 MBq of [^18^F]FLUTE (90–110 min), which was purchased from Carecamp Inc. A low-dose CT was performed for attenuation correction prior to all scans. In participants who underwent [^18^F]THK5351 and [^18^F]FLUTE PET scans, the mean intervals between THK PET and FLUTE PET scans were 10 days. FLUTE PET and MRI scans were acquired on the same day. Individual static images were reconstructed onto a 256 × 256 × 109 matrix with a voxel size of 1.3 × 1.3 × 1.5 mm^3^ using a 2D Ordered Subset Expectation Maximization (OSEM) algorithm (eight iterations and 16 subsets), with corrections for physical effects.

#### PET Quantification

Each [^18^F]THK5351 and [^18^F]FLUTE PET image was co-registered onto individual T1 images using FreeSurfer. Region-based partial volume correction (PVC) was performed on the regional mean values of PET images using the PETSurfer tool in FreeSurfer (Greve et al., 2014, 2016). We defined 18 regions of interest (ROIs) to compare [^18^F]THK5351 retention in each group quantitatively including the prefrontal (frontal pole, pars orbitalis, orbital frontal, pars triagularis, pars opercularis, rostral middle frontal, superior frontal, caudal middle frontal, and medial orbital frontal regions), orbitofrontal (orbital frontal and medial orbital frontal regions), sensorimotor (pre-central, post-central, and paracentral regions), anterior cingulate (accumbens-area, caudal anterior cingulate, and rostral anterior cingulate regions), superior parietal, inferior parietal (inferior parietal and supramarginal regions), pre-cuneus, posterior cingulate, occipital cortex (pre-cuneus, pericalcarine, and lateral occipital regions), superior temporal, middle temporal, inferior temporal, mesial temporal (hippocampus, amygdala, parahippocampal and entorhinal cortices), entorhinal cortices, parahippocampus, fusiform gyrus, lingual gyrus, and global cortex (a composition of the prefrontal, superior parietal, lateral temporal, inferior parietal, occipital, anterior cingulate, mesial temporal pre-cuneus, and posterior cingulate cortices).

Regional standardized uptake value ratios (SUVRs) were calculated with reference to the cerebellar gray matter for THK images (Okamura et al., 2014; Lockhart et al., 2016), as the cerebellar gray matter is the one of the least affected areas in AD and there is no difference in MAO-B density of the cerebellum between healthy controls and AD patients (Saura et al., 1994). According to a previous study with FLUTE PET, which showed diagnostic accuracy was most excellent when using pons as the reference region, we also used the pons for FLUTE images (Thurfjell et al., 2014). SUVR images were also generated from the MRI co-registered PET images with voxel-based PVC (Okamura et al., 2014; Lockhart et al., 2016). The FLUTE cortex retention ratio was calculated based on AD-related regions including the frontal, parietal, lateral temporal, anterior, and posterior cingulate cortices (Thurfjell et al., 2014). An SUVR threshold of 0.62 for amyloid positivity was applied to the [^18^F]FLUTE PET data (Thurfjell et al., 2014).

### Cluster Analyses

**Figure 1:**
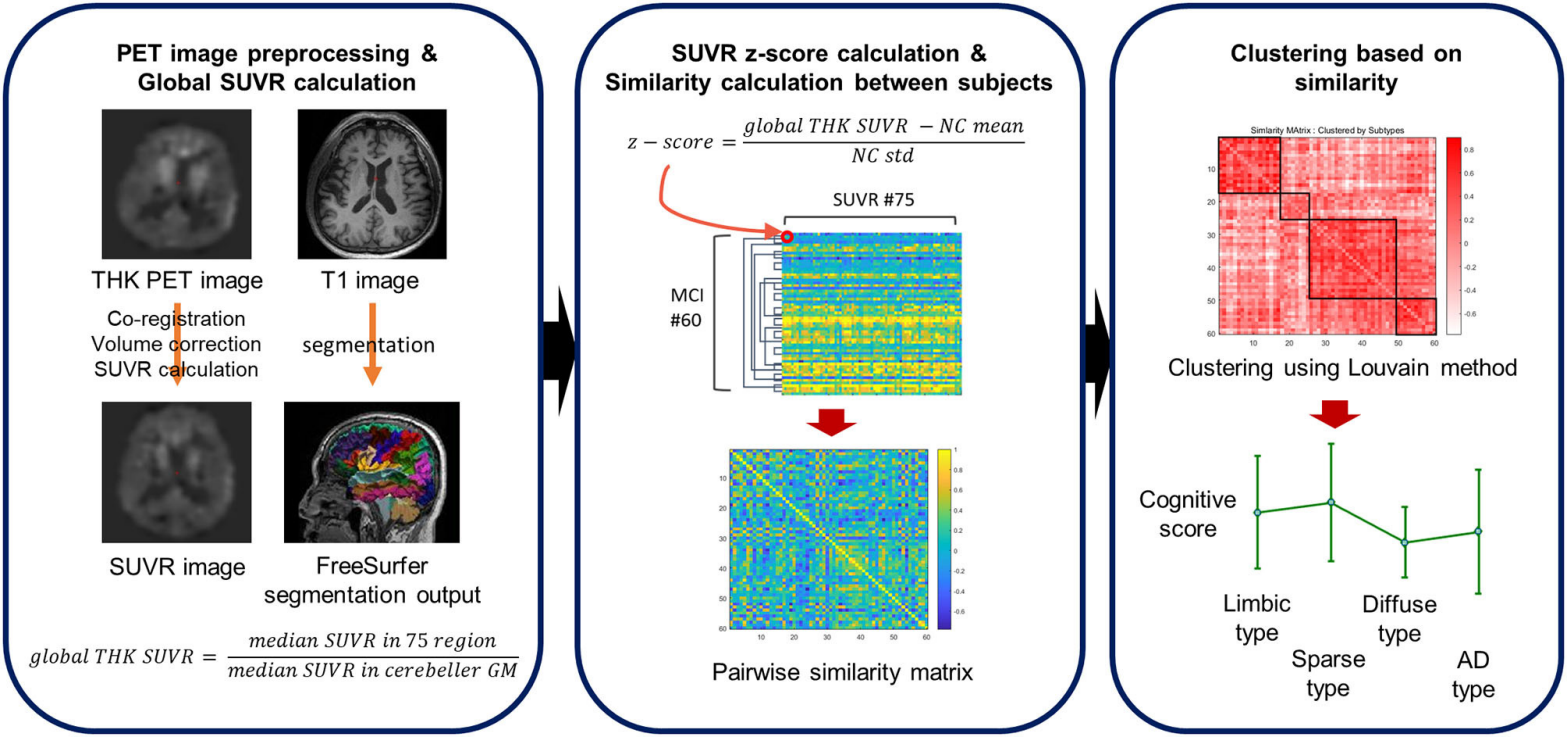
A schematic overview of the clustering pipeline. Z-scores are computed for each subject's THK SUVR with respect to the cognitively normal (CN) subjects. The similarity of any pair of subjects is defined using a correlation coefficient between regional THK SUVR levels of the subjects.

Before dividing MCI patients into subtypes using the degree of cortical uptake of THK and subcortical regions (total of 75 regions), we calculated z-scores of those values using 63 NC subjects' global SUVR values. Standardized SUVR values were extracted for each of the 75 ROIs for each MCI patient. To calculate the degree of similarity between each pair of MCI patients, the correlation matrix was computed. Depending on the pairwise correlation, MCI patients were then clustered using the Louvain method (29, 30). The overall process is depicted in Figure 1.

The Louvain method is a greedy optimization method used to extract clusters based on modularity in network science. The Louvain clustering method has two major steps that are conducted iteratively. In the first stage, we treated each MCI subject as a node and generated a graph with correlation coefficients as edges. Initially, each subject was assigned to an individual group, so the initial number of clusters was the same as the number of MCI patients. As the iterations proceeded, the MCI patients with higher correlations are assigned to the same cluster so that the modularity of the graph is maximized. However, due to its greedy approach, the clustering results can vary. Therefore, we performed the clustering multiple times, before each node was assigned based on a major voting scheme in the second stage. Since the cluster information for each MCI patient was obtained after several greedy optimizations, the method has high reproducibility and reliability.

In this process, the resolution parameter, the gamma value (which controls the number of clusters) was used. Different gamma values from 0.7 to 1.0 were applied and gamma set such that the resulting clusters were subdivided into different THK retention patterns with high modularity. Sixty MCI patients were clustered into four subtypes after 1,000 iterations. Based on the results, the patients' neuropsychological test scores were compared between subtypes to assess how the neuropsychological scores differed between the groups.

### Definition of Dementia at Follow-up

Dementia conversion was evaluated from February to April 2019 through electronic medical record documents and interviews with patients and caregivers. The median duration of follow-up was 34.6 months (IQR: 23.1–40.5 months). Dementia was diagnosed according to the diagnostic algorithm detailed in Structured Interview for Diagnosis of Dementia of Alzheimer's type, Multi-infarct Dementia and Dementia of other Etiology according to the Diagnostic and Statistical Manual of Mental Disorders, version IV (DSM-IV) and the International Classification of Diseases, version 10 (ICD-10) (SIDAM) (Jessen et al., 2014). Diagnosis of AD was made on the basis of criteria for probable AD proposed by the National Institute of Neurological and Communicative Disorders and Stroke–Alzheimer's Disease and Related Disorders Association (NINCDS-ADRDA) (McKhann et al., 1984). Dementia with Lewy bodies was diagnosed according to the Fourth Consensus Report of the DLB consortium (McKeith et al., 2017). Subcortical vascular dementia (SVaD) was diagnosed according to DSM-IV criteria for VaD and severe white matter hyperintensities (WMH) on MRI. Severe WMH on MRI was defined as a cap or a band ≥10 mm as well as a deep white matter lesion ≥25 mm, as modified from Fazekas ischemia criteria (Yoon et al., 2013).

### Statistical Analyses

The Kruskal-Wallis test was used to compare the demographic and clinical data, regional THK SUVRs, and neuropsychological test results between all five groups (Limbic, Diffuse, Sparse, AD type, and NC group). *Post-hoc* analysis using the Mann-Whitney U test was performed to determine the significance of the differences between pairs of groups for continuous variables, and Fisher's exact test was used to compare the distribution of categorical variables. To ensure that the likelihood of making a type I error remained at <5% (<0.05), we applied Sidak-adjusted *P*-values to the Mann-Whitney test results, in which a null hypothesis was considered rejected when *P* < 0.005. All of the above statistical analyses were conducted using SPSS (IBM SPSS statistics version 24; SPSS Inc., Chicago, IL, USA). Voxel-wise analyses were performed to compare regional patterns of [^18^F]THK5351 retention using SPM12 (Statistical Parametric Mapping; Wellcome Trust Center for Neuroimaging, London, UK). For the analysis of comparisons of SUVR images, we performed 2-sample *t*-tests with adjustments for age and years of education. The odds ratio (OR) of conversion to dementia in each MCI subgroup was estimated using Firth's logistic regression controlling for age, gender, educational year, and baseline date, as we set the reference group to a subgroup with zero subjects progressing to dementia. Statistical analysis was performed with SAS version 9.4.

## Results

### MCI Subgroups Identified by Cluster Analyses

**Figure 2:**
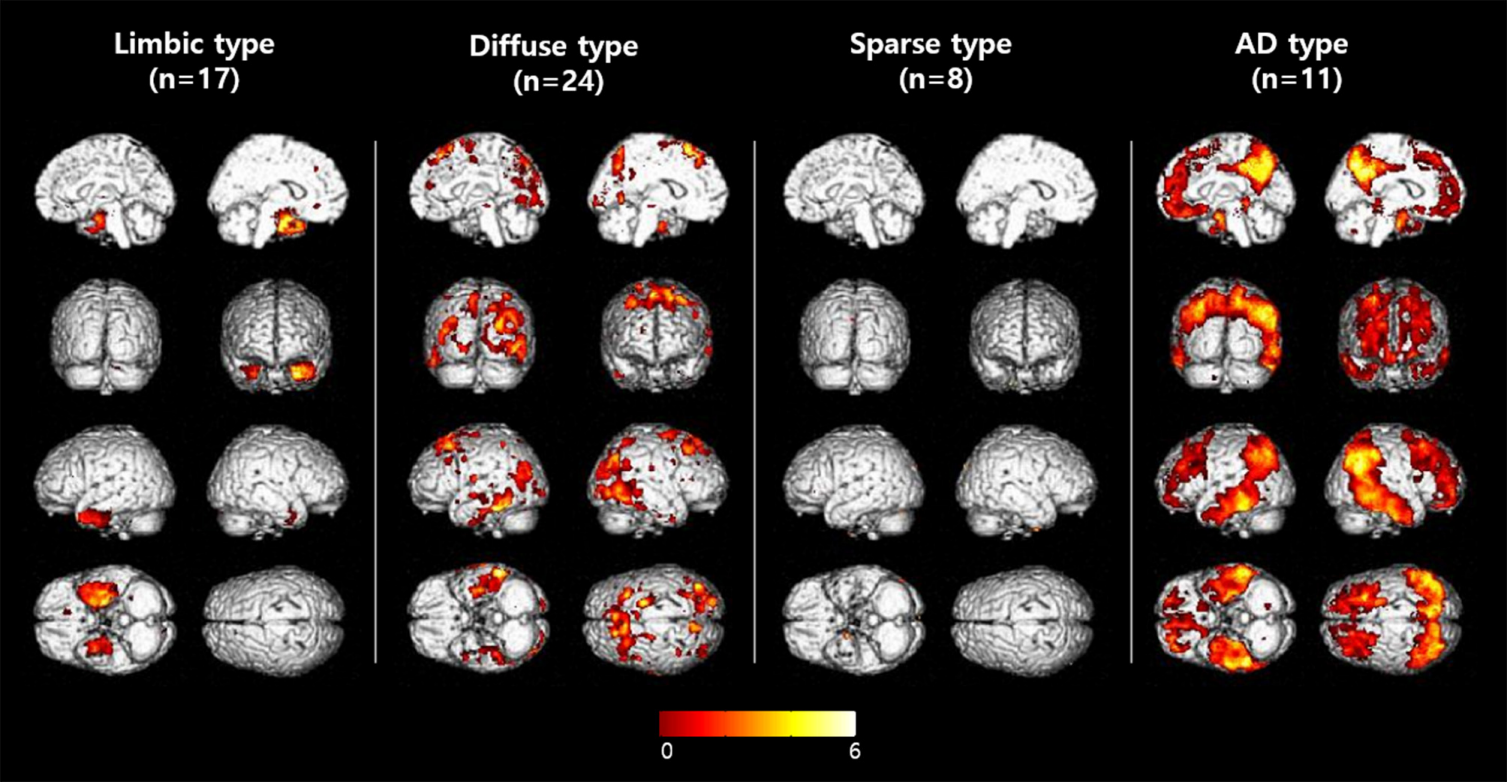
Voxelwise comparisons of [^18^F]THK5351 retention among MCI subtypes. Voxelwise comparisons of [^18^F]THK5351 retention between each MCI group to the control group (uncorrected for multiple comparisons with adjustment for age and years of education at a threshold of *p* < 0.001).

MCI patients were categorized into one of four groups according to the [^18^F]THK5351 retention patterns compared to NC: (1) limbic predominant retention (limbic type, *n* = 17); (2) diffuse retention (diffuse type, *n* = 24); (3) sparse retention (sparse type, *n* = 8); or (4) retention of AD pattern (AD type, *n* = 11) (Figure 2, Table 1).

**Table 1:** Regional SUVR values from [^18^F] THK5351 PET scans of MCI patient subtypes and controls.

**ROIs**	**Limbic type** **(*n* = 17)**	**Diffuse type** **(*n* = 24)**	**Sparse type** **(*n* = 8)**	**AD type** **(*n* = 11)**	**NC** **(*n* = 37)**	*p-*Value^*^
Prefrontal	1.74 (1.29, 1.89)	1.75 (1.51, 2.06)^a^	1.45 (1.34, 1.69)	1.82 (1.67, 1.99)^a^	1.47 (1.31, 1.69)^c^^,^ ^e^	*p* = 0.001
Orbitofrontal	2.29 (1.73, 2.69)	2.14 (1.90, 2.56)	1.92 (1.75, 2.30)	2.36 (2.17, 2.52)^a^	1.92 (1.69, 2.16)^e^	*p* = 0.010
Sensorimotor	1.19 (0.97, 1.39)	1.28 (1.16, 1.51)	1.15 (1.09, 1.27)	1.21 (1.16, 1.33)	1.14 (1.02, 1.30)	*p* = 0.077
Anterior cingulate	3.14 (2.92, 3.62)	3.07 (2.70, 3.44)	3.19 (2.85, 3.41)	3.21 (2.92, 3.41)	2.97 (2.58, 3.30)	*p* = 0.224
Superior parietal	1.27 (1.04, 1.41)	1.60 (1.28, 1.87)^a^	1.38 (1.22, 1.54)	1.57 (1.50, 1.80)^a^	1.27 (1.13, 1.39)^c^^,^ ^e^	*p* < 0.001
Inferior parietal	1.61 (1.27, 1.85)	1.83 (1.44, 2.04)^a^	1.46 (1.27, 1.61)	1.91 (1.64, 2.26)^a^	1.40 (1.22, 1.57)^c^^,^ ^e^	*p* < 0.001
Pre-cuneus	1.71 (1.26, 1.93)	1.85 (1.55, 2.16)^a^	1.62 (1.41, 1.78)^e^	2.01 (1.84, 2.79)^a^^,^ ^d^	1.51 (1.34, 1.69)^c^^,^ ^e^	*p* < 0.001
Posterior cingulate	2.03 (1.81, 2.54)	2.17 (1.86, 2.54)	1.99 (1.79, 2.21)	2.50 (2.16, 2.80)^a^	1.96 (1.72, 2.12)^e^	*p* = 0.002
Occipital	1.19 (1.01, 1.34)	1.40 (1.14, 1.67)^a^	1.24 (1.01, 1.36)	1.21 (1.10, 1.28)	1.15 (0.96, 1.22)^c^	*p* = 0.002
Superior temporal	2.06 (1.61, 2.22)	1.93 (1.68, 2.21)	1.76 (1.51, 1.88)	1.96 (1.67, 2.48)	1.73 (1.44, 1.91)	*p* = 0.014
Middle temporal	1.92 (1.64, 2.32)	2.14 (1.76, 2.44)^a^	1.78 (1.38, 2.04)	2.23 (1.86, 2.56)^a^	1.66 (1.51, 1.93)^c^^,^ ^e^	*p* < 0.001
Inferior temporal	1.99 (1.58, 2.34)	2.13 (1.72, 2.38)^a^	1.79 (1.44, 1.93)	2.17 (1.92, 2.35)^a^	1.62 (1.48, 1.85)^c^^,^ ^e^	*p* < 0.001
Mesial temporal	3.30 (2.68, 3.86)^a^	2.78 (2.29, 3.00)	2.66 (2.47, 2.91)	3.30 (2.71, 3.48)^a^	2.46 (2.26, 2.68)^b^^,^ ^e^	*p* < 0.001
Entorhinal	2.96 (2.61, 3.62)^a^^,^ ^d^	2.37 (2.08, 2.77)^b^	2.35 (2.10, 2.52)^b^	2.79 (2.39, 2.92)^a^	2.19 (1.97, 2.41)^b^^,^ ^e^	*p* < 0.001
Parahippocampus	2.21 (1.97, 2.80)	2.21 (1.94, 2.50)	2.08 (1.99, 2.38)	2.48 (2.09, 2.85)^a^	1.96 (1.76, 2.21)^e^	*p* = 0.004
Fusiform gyrus	1.85 (1.63, 2.00)^a^	1.91 (1.61, 2.23)^a^	1.70 (1.51, 1.84)	1.92 (1.74, 2.26)^a^	1.54 (1.39, 1.77)^b^^,^ ^c^^,^ ^e^	*p* < 0.001
Lingual gyrus	1.35 (1.09, 1.44)	1.47 (1.22, 1.65)^a^	1.42 (1.28, 1.50)	1.33 (1.31, 1.52)	1.26 (1.06, 1.39)^c^	*p* = 0.007
Global cortex	1.84 (1.44, 2.04)	1.90 (1.56, 2.17)^a^	1.63 (1.44, 1.82)	2.02 (1.74, 2.11)^a^	1.57 (1.38, 1.76)^c^^,^ ^e^	*p* = 0.001

Data are presented as median (interquartile range) for continuous variables.

* P-values were determined using Kruskal-Wallis test across groups.

a Mann-Whitney U tests, Sidak-adjusted p < 0.005 vs. NC.

b Mann-Whitney U tests, Sidak-adjusted p < 0.005 vs. Limbic type.

c Mann-Whitney U tests, Sidak-adjusted p < 0.005 vs. Diffuse type.

d Mann-Whitney U tests, Sidak-adjusted p < 0.005 vs. Sparse type.

e Mann-Whitney U tests, Sidak-adjusted p < 0.005 vs. AD type.

*MCI, mild cognitive impairment; NC, normal control; PET, positron emission tomography; ROI, region of interest; SUVR, standardized uptake value ratio*.

In patients of the limbic type, [^18^F]THK5351 retention was significantly greater than the NC group in the mesial temporal, entorhinal cortices, and fusiform gyrus areas.

In diffuse type patients, [^18^F]THK5351 retention was significantly greater than in the NC group in the prefrontal, superior and inferior parietal areas, pre-cuneus, occipital, middle and inferior temporal, fusiform gyrus, lingual gyrus, and global cortex.

ROI-based analyses showed that patients of the sparse type did not have significantly greater [^18^F]THK5351 retention than the NC group in any ROI areas. Moreover, retention in the sparse type was lower than in the other MCI subtypes, although not statistically significant, in 16 ROIs (prefrontal, orbitofrontal, sensorimotor, inferior parietal, pre-cuneus, anterior cingulate, posterior cingulate, occipital, superior temporal, middle temporal, inferior temporal, mesial temporal, entorhinal cortices, parahippocampus, fusiform gyrus, and global cortex).

In AD type patients, [^18^F]THK5351 retention was significantly greater than the NC groups in 14 ROIs including prefrontal, orbitofrontal, superior parietal and inferior parietal areas, pre-cuneus, posterior cingulate, middle temporal, inferior temporal, mesial temporal, and entorhinal cortices, parahippocampus, fusiform gyrus, and global cortex (Table 1).

### Baseline Demographic and Clinical Characteristics

Detailed demographic and clinical characteristics of the study population are presented in Table 2.

**Table 2:** Demographics and clinical characteristics of the study population.

	**Limbic type** **(*n* = 17)**	**Diffuse type** **(*n* = 24)**	**Sparse type** **(*n* = 8)**	**AD type** **(*n* = 11)**	**NC** **(*n* = 37)**	*p-*Value^*^
Baseline age (years)	76.0 (63.0, 79.0)	71.5 (65.25, 77.0)	63.5 (52.0, 70.0)	67.0 (64.0, 73.0)	65.0 (54.5, 74.0)	*p* = 0.008
Age at onset (years)	73.0 (59.5, 76.5)	68.5 (63.5, 74.5)	60.0 (48.7, 66.5)	65.0 (59.0, 69.0)	–	*p* = 0.044
Gender (female, *n* [%])	11 (64.7%)	16 (66.7%)	5 (62.5%)	8 (72.7%)	20(54.1%)	*p* = 0.792
Education (years)	6.0 (3.0, 10.5)^a^	6.5 (6.0, 12.0)^a^	10.5	9.0 (9.0, 16.0)	12.0^b^^,^ ^c^	*p* = 0.003
Disease duration (months)	36.0 (33.0, 48.0)	24.0 (13.0, 42.0)	36.0 (12.0, 46.5)	36.0 (24.0, 48.0)	–	*p* = 0.198
MMSE	24.0 (20.5, 27.0)^a^	26.5 (24.0, 28.0)^a^	26.5 (24.5, 28.0)	22.0 (17.0, 26.0)^a^	29.0 (27.5, 30.0)^b^^,^ ^c^^,^ ^e^	*p<0.001*
CDR SOB	1.0 (0.5, 1.75)^a^	0.5 (0.5, 1.38)^a^^,^ ^e^	0.5 (0.5, 0.5)^a^^,^ ^e^	2.0 (1.5, 3.0)^a^^,^ ^b^^,^ ^c^^,^ ^d^	0.0 (0, 0)^b^^,^ ^c^^,^ ^d^^,^ ^e^	*p<0.001*
APOE ε4 (carrier, *n* [%])	6/17 (35.3%)	7/24 (29.2%)	1/8 (12.5%)	7/11 (63.6%)	8/37 (21.6%)	*p* = 0.090
Amyloid positivity (%)	4/7 (57.1%)	4/8 (50.0%)	0/1 (0%)	6/9 (66.7%)	0/37 (0%)	*p<0.001*
aMCI (*n* [%])	8 (47.1%)	17 (70.8%)	4 (50.0%)	10 (90.9%)	–	*p* = 0.070
aMCI multiple domain (*n* [%])	7/17 (41.2%)	14/24 (58.3%)	3/8 (37.5%)	9/11 (81.8%)	–	*p* = 0.479
Hypertension	9 (52.9%)	14 (58.3%)	3 (37.5%)	4 (36.4%)	12 (32.4%)	*p* = 0.294
Diabetes mellitus	5 (29.4%)	4 (16.7%)	0 (0%)	1 (9.1%)	3 (8.1%)	*p* = 0.220
Coronary artery disease	2 (11.8%)	5 (20.8%)	0 (0%)	0 (0%)	3 (8.1%)	*p* = 0.371
Dyslipidemia	7 (41.2%)	9 (37.5%)	1 (12.5%)	4 (36.4%)	13 (35.1%)	*p* = 0.734
History of stroke	3 (17.6)	2 (8.3%)	0 (0%)	1 (9.1%)	0 (0%)	*p* = 0.068
Total lacunes	1.0 (0.0, 2.0)	1.0 (0.0, 1.75)	0.0 (0.0, 0.75)	0.0 (0.0, 2.0)	0.0 (0.0, 1.0)	*p* = 0.191
Total microbleeds	0.0 (0.0, 0.0)	0.0 (0, 0)	0.0 (0, 0)	0.0 (0, 0)	0.0 (0, 0)	*p* = 0.544
Total WMH volume (mm^3^)	2488.0 (1863.50, 6812.50)	6234.5 (3074.25, 11966.0)^a^	1797.0 (1068.0, 3404.5)	3382.0 (2054.0, 7120.0)	1930.0 (1352.5, 3169.0)^c^	*p* < 0.001
PWMH volume (mm^3^)	2464.0 (1692.0, 5863.00)	4929.0 (2832.5, 11180.75)^a^	1592.0 (1005.5, 3144.5)	2819.0 (1891.0, 5953.0)	1746.0 (1206.5, 2891.5)^c^	*p* < 0.001
DWMH volume (mm^3^)	235.0 (99.0, 663.5)	386.5 (181.75, 1427.0)	168.0 (53.0, 288.0)	267.0 (163.0, 582.0)	139.0 (47.0, 369.0)	*p* = 0.079
Mean CTh (mm)	2.46 (2.34, 2.54)	2.46 (2.38, 2.63)	2.52 (2.45, 2.61)	2.42 (2.26, 2.51)	2.48 (2.4, 2.6)	*p* = 0.309
Hippocampal volume (mm^3^)	3306.70 (2950.58, 3768.58)^a^	3585.45 (3354.5, 3848.9)^a^	3915.78 (3622.09, 4460.76)	3177.35 (2917.0, 3724.0)^a^	3955.75 (3693.58, 4270.7)^b^^,^ ^c^^,^ ^e^	*p* < 0.001
ICV (mm^3^)	1378414.48 (1281901.52, 1502008.65)	1415790.01 (1240549.67, 1624775.24)	1315494.11 (1134301.72, 1615072.3)	1417803.95 (1239112.12, 1508740.82)	1351686.70 (1260247.34, 1491808.13)	*p* = 0.875

Data are presented as median (interquartile range) for continuous variables and number (%) for nominal variables.

The variables age of onset, disease duration, aMCI ratio, and aMCI multiple domain ratio were compared except for NC, and all other variables were compared among the five groups including NC.

Fisher's exact test used for nominal variables.

* P-values were determined using Kruskal-Wallis test across groups except nominal variables.

a Mann-Whitney U tests, Sidak-adjusted p < 0.005 vs. NC.

b Mann-Whitney U tests, Sidak-adjusted p < 0.005 vs. Limbic type.

c Mann-Whitney U tests, Sidak-adjusted p < 0.005 vs. Diffuse type.

d Mann-Whitney U tests, Sidak-adjusted p < 0.005 vs. Sparse type.

e Mann-Whitney U tests, Sidak-adjusted p < 0.005 vs. AD type.

*aMCI, amnestic mild cognitive impairment; CDR SOB, clinical dementia rating–sum of boxes; CTh, cortical thickness; DWMH, deep white matter hyperintensity; ICV, intracranial volumes; MMSE, mini-mental state examination; NC, normal control; PWMH, periventricular white matter hyperintensity; WMH, white matter hyperintensity*.

Patients classified as limbic type were old with a median age of 76.0. These patients had an APOE e4 carrier ratio of 35.3% (*n* = 6/17), amyloid positivity of 57.1% (*n* = 4/7), and aMCI prevalence (multiple domain) of 41.2% (*n* = 7/17). Hippocampal volumes were significantly lower than in those the control group.

Diffuse type patients had a median age of 71.5. These patients had an APOE e4 carrier ratio of 29.2% (*n* = 7/24), amyloid positivity of 57.1% (*n* = 4/7), and aMCI prevalence (multiple domain) of 41.2% (*n* = 7/17). They had significantly larger total WMH and periventricular white matter hyperintensity (PWMH) volumes than the controls. Hippocampal volumes were also significantly lower than those in the controls.

The average age of the sparse type patients (median age, 63.5 years; range, 52–70) was the youngest among the five groups, including the control group (median age, 65 years; range, 54.5–74.0). Their CDR-SOB scores were significantly worse than those of the control group, but better than those of the AD type group. These patients also had the lowest proportion APOE e4 carriage (12.5%, *n* = 1/8), amyloid positivity (0%, *n* = 0/8), and aMCI prevalence (multiple domain) (37.5%, *n* = 3/8) among the four MCI subtypes.

The AD type had the worst MMSE (median score, 22; range, 17–26) and CDR-SOB scores (median score, 2.0; range 1.5–3.0) among the MCI groups, which were significantly different from the control group as well as the other MCI groups. Furthermore, they had the highest proportion APOE e4 carriage (63.6%, *n* = 7/11), aMCI (90.9%, *n* = 10/11), and aMCI multiple domain prevalence (81.8%, *n* = 9/11) among the four MCI subtypes. Mean cortical thickness (CTh) in the AD type appeared smaller than the other groups, but there was no significant difference. Meanwhile, hippocampal volumes in the AD type were significantly smaller than the controls.

### Neuropsychological Test Results Comparison

Neuropsychological test results showed statistical significance for all tests except the visuospatial function test (RCFT). The detailed results are presented in Table 3. For comparison of the neuropsychological test results, we used age- and education-adjusted standard scores (*z* scores).

**Table 3:** Neuropsychological test results.

	**Limbic type** **(*n* = 17)**	**Diffuse type** **(*n* = 24)**	**Sparse type** **(*n* = 8)**	**AD type** **(*n* = 11)**	**NC** **(*n* = 37)**	*p-*Value^*^
**ATTENTION**						
Digit span test: forward	0.15 (−0.49, 1.33)	0.51 (−0.39, 1.57)	−0.18 (−0.83, 0.46)^a^	−0.64 (−1.16, 1.28)	1.22 (0.73, 1.51)^d^	*p* = 0.006
Digit span test: backward	−0.28 (−0.77, 0.39)	−0.19 (−0.59, 0.48)	−0.32 (−1.19, −0.09)	0.07 (−1.62, 0.57)	0.22 (−0.36, 0.96)	*p* = 0.057
**LANGUAGE FUNCTION**						
K-BNT	−0.12 (−0.76, 0.17)^e^	−0.31 (−1.02, 0.23)^e^	−0.64 (−1.62, 0.02)	−2.40 (−3.33, −1.48)^a^^,^ ^b^^,^ ^c^	0.15 (−0.49, 0.85)^e^	*p* < 0.001
**VISUOSPATIAL FUNCTION**						
RCFT copy	0.66 (−1.00, 1.01)	0.18 (−0.91, 0.97)	0.69 (−1.16, 1.03)	0.05 (−1.14, 0.93)	0.57 (0.15, 1.00)	*p* = 0.393
**MEMORY**						
SVLT, immediate recall	−0.61 (−1.49, −0.09)^a^	−0.44 (−1.15, 0.20)^a^^,^ ^e^	−0.90 (−1.16, 0.28)	−1.42 (−2.35, −0.84)^a^^,^ ^c^	0.46 (−0.24, 0.75)^b^^,^ ^c^^,^ ^e^	*p* < 0.001
SVLT, delayed recall	−0.77 (−1.65, 0.01)^a^^,^ ^e^	−0.66 (−1.66, 0.05)^a^^.^^e^	−0.23 (−1.16, 0.12)^e^	−2.30 (−2.96, −2.09)^a^^,^ ^b^^,^ ^c^^,^ ^d^	0.33 (−0.11, 0.98)^b^^,^ ^c^^,^ ^e^	*p* < 0.001
RCFT, immediate recall	−0.23 (−0.69, 1.11)^e^	−0.21 (−1.22, 0.26)^a^^,^ ^e^	−0.38 (−0.79, 0.52)	−1.50 (−2.06, −1.11)^a^^,^ ^b^^,^ ^c^	1.07 (0.09, 1.49)^c^^,^ ^e^	*p* < 0.001
RCFT, delayed recall	−0.39 (−1.19, 0.88)^a^^,^ ^e^	−0.81 (−1.51, 0.03)^a^	−0.50 (−1.33, 0.35)^a^^.^^e^	−1.61 (−2.45, −1.07)^a^^,^ ^b^^,^ ^d^	0.96 (0.19, 1.55)^b^^,^ ^c^^,^ ^d^^,^ ^e^	*p* < 0.001
**FRONTAL/EXECUTIVE FUNCTION**						
COWAT, animal	−0.93 (−1.49, −0.11)^a^^,^ ^e^	−0.87 (−1.24, −0.47)^a^^,^ ^e^	−0.76 (−1.03, −0.02)	−2.23 (−2.48, −1.39)^a^^,^ ^b^^,^ ^c^	−0.03 (−0.59, 0.79)^b^^,^ ^c^^,^ ^e^	*p* < 0.001
COWAT, supermarket	−0.53 (−1.35, 0.33)	−0.71 (−1.18, 0.03)^a^	−0.67 (−0.94, 0.25)	−1.32 (−1.74, −0.65)^a^	0.24 (−0.42, 1.18)^c^^,^ ^e^	*p* < 0.001
COWAT, phonemic fluency	−0.30 (−1.02, 0.99)	−0.40 (−1.22, 0.53)	−0.33 (−0.82, 0.71)	−0.75 (−1.60, 0.34)	0.68 (0.08, 1.08)	*p* = 0.010
Stroop test, color reading	−0.40 (−1.78, 0.25)	−0.25 (−1.10, 0.79)	−0.45 (−1.68, 0.01)	−1.27 (−2.88, −0.30)^a^	0.35 (−0.35, 0.94)^e^	*p* < 0.001
TMT-B	−1.21 (−2.92, 0.49)^e^	−0.53 (−1.30, 0.60)^e^	−1.26 (−1.62, 0.71)	−5.95 (−8.49, −1.48)^a^^,^ ^b^^,^ ^c^	0.39 (−0.33, 0.91)^e^	*p* < 0.001

Data are presented as median (interquartile range) for continuous variables. All data are z-scores.

* P-values were determined using Kruskal-Wallis test across groups.

a Mann-Whitney U tests, Sidak-adjusted p < 0.005 vs. NC.

b Mann-Whitney U tests, Sidak-adjusted p < 0.005 vs. Limbic type.

c Mann-Whitney U tests, Sidak-adjusted p < 0.005 vs. Diffuse type.

d Mann-Whitney U tests, Sidak-adjusted p < 0.005 vs. Sparse type.

e Mann-Whitney U tests, Sidak-adjusted p < 0.005 vs. AD type.

aMCI, amnestic mild cognitive impairment; CDR SOB, clinical dementia rating–sum of boxes; CTh, cortical thickness; DWMH, deep white matter hyperintensity; ICV, intracranial volumes; MMSE, mini-mental state examination; NC, normal control; PWMH, periventricular white matter hyperintensity; WMH, white matter hyperintensity.

*COWAT, Controlled Oral Word Association Test; K-BNT, Korean version of the Boston Naming Test; NC, normal control; RCFT, Rey–Osterrieth Complex Figure Test; SVLT, Seoul Verbal Learning Test; TMT-B, Trail Making Test Part B*.

Patients of the limbic type showed reduced in three tests in memory function and a test in frontal/executive function. Diffuse type patients showed reduced verbal and visual memory and frontal/executive functions. Sparse type patients exhibited relatively preserved cognitive functions. The patients of this group just showed reduced function in attention (digit span forward) and visual memory (RCFT-DR). Scores for the AD type patients were significantly worse for cognitive functions including language, memory, and frontal/executive functions. Most of the test results were worse than for the other MCI subtypes (Table 3).

### Conversion to Dementia

Of the 60 patients with MCI, two patients of the diffuse type were lost to follow-up. One patient passed away from pancreatic cancer 5 months after the baseline date, and the other was lost to follow-up after a significant intracerebral hemorrhage (ICH) 5 months after the baseline date. Eighteen of the 58 MCI patients progressed to dementia (31.0%) and there was a median duration of follow-up of 34 months. Firth's logistic regression analyses showed that the OR of conversion to dementia was higher in the AD type (rate of conversion = 90.9%, OR = 88.95, 95% CI = 3.224–>999.9, *p* = 0.008) than in sparse type patients after controlling for age, gender, educational years, and follow-up interval from baseline date (Table 4). The OR for the limbic and diffuse type was not significant. Of the 18 patients that converted to dementia, 16 patients progressed to probable AD dementia [3 (17.6%) of the limbic type, 5 (22.7%) of the diffuse type, 8 (72.7%) of the AD type], 1 patient in the AD type progressed to probable dementia with Lewy bodies (DLB), and one patient in the AD type had subcortical vascular dementia. During the follow-up period, some patients were newly diagnosed with other diseases: 1 patient in the limbic type developed pancreatic cancer and three patients in the diffuse type developed hepatocellular carcinoma, pancreatic cancer and intracerebral hemorrhage, respectively.

**Table 4:** Conversion to dementia in each MCI subgroup.

	***n***	**Number of subjects progressing to dementia**	**OR**	**95% CI**	***p-*Value**
Sparse type	8	0 (0%)	1	Reference	
Limbic type	17	3 (17.6%)	2.980	0.132–67.18	0.492
Diffuse type	22	5 (22.7%)	4.479	0.224–89.45	0.326
AD type	11	10 (90.9%)	88.95	3.224–>999.9	0.008

Data was generated using Firth's logistic regression analyses for progression to dementia after controlling for age, gender, educational years, and baseline date.

*OR, odds ratio; CI, confidence interval*.

## Discussion

Our major finding is that significant differences in topographical patterns of [^18^F]THK5351 retention which detects reactive astrogliosis and tau, can be used to distinguish MCI subtypes. Cluster analysis based on [^18^F]THK5351 retention patterns showed that MCI patients can be categorized distinctly according to anatomical differences. There were also differences in demographic and clinical characteristics as well as risk of progression to dementia among the subtypes in accordance with differences in THK5351 retention.

A recent study reported that the presence of a MAO-B inhibitor reduced THK 5351 binding and showed that THK5351 can also bind to MAO-B across the whole brain (Ng et al., 2017). This finding has been supported by another recent study (Harada et al., 2018) reporting that THK binding may reflect a mixture of pathologic tau and astrocytes with abundant MAO-B. MAO-B off target binding may limit the interpretation of THK5351 retention regarding its reflection of genuine pathologic NFT. However, MCI is a heterogeneous disease entity that may be caused by various etiologies which may lead to diverse prognosis. [^18^F]THK5351 PET may be used for differential diagnosis of neurodegenerative diseases using topographical characteristics which reflect clinically corresponding brain areas (Brendel et al., 2017; Ishiki et al., 2017; Lee et al., 2018; Schönecker et al., 2019; Son et al., 2019) and is suitable to detect neurodegenerative changes not limited to NFT.

Among the four subtypes, patients of the limbic type in which [^18^F]THK5351 retention elevated primarily in the mesial temporal and entorhinal regions showed distinct demographic and clinical features. They were older (median age, 76.0) at onset and had smaller hippocampal volumes. Limbic type patients had mainly memory declines in neuropsychological tests, and OR for conversion to dementia during the follow-up period (17.6%, *n* = 3/17) was lower than that of AD type (90.9%, *n* = 10/11). Therefore, if patients in this group progress to dementia at a later time, they are more likely to progress to the limbic predominant type (Murray et al., 2011) and medial temporal type (Noh et al., 2014) according to previous cluster analysis studies performed using neuropathology and MRI. It remains to be determined because the pathology was not confirmed in the previous study, but it appears that the limbic type may be a limbic-predominant age-related TDP-43 encephalopathy (LATE), as revealed in recent studies (Nelson et al., 2019).

Patients in diffuse type exhibited diffuse retention throughout the cerebral cortices including prefrontal, parietal, lateral temporal, and occipital areas. However, regional SUVR of the mesial temporal cortex was not greater than NC, while limbic type or AD type showed greater THK retention in this region. Median onset age was 68.5 (IQR 63.5–74.5) and it was older than AD type although the difference was not statistically significant. In a previous pathologic study, AD patients of the diffuse type based on topography of NFT deposition were more likely to typical late onset AD clinically (Murray et al., 2011). Cluster analysis study based on cortical thickness using MRI showed the AD dementia patients in diffuse atrophy subtype were older, presented with typical AD symptoms and had thinner cortical thickness (Noh et al., 2014). Late onset AD has known to have more mixed pathologies including vascular pathololgies and degenerative pathologies other than amyloid or NFT than in early onset AD (De Ture and Dickson, 2019). White matter hyperintensities were larger in the diffuse type, which may contribute to cognitive functions or neurodegeneration in this group. Considering a recent report that elevated flortaucipir retention was observed in patients with subcortical vascular cognitive impairment (SVCI) (Kim et al., 2018), some pathologic changes, such as astrogliosis might contribute to elevated THK5351 retention in subjects with vascular burden. Meanwhile, the patients in diffuse type showed higher comorbidity and mortality during follow-up period than those in other groups. Not long after the baseline study, cancer was diagnosed in two of the 24 patients, one of whom died and another experienced an intracerebral hemorrhage.

Patients of the sparse type were diagnosed with MCI on history and cognitive function tests, but none of them progressed to dementia during follow-up period. Patients in the sparse group had a lower age at onset (median age, 63.5; range 50.5–70.0) and neuropsychiatric tests revealed only a reduction in attention and visual memory. Therefore, this group seems to be comprised of individuals whose cognitive functions were deteriorated temporarily by other causes, such as psychiatric problems, rather than by degenerative pathologies.

The AD type patients were younger than the limbic and diffuse type, although the difference was not significant. At baseline, disease duration was not different with other groups, but cognitive function was the poorest. Although not statistically significant, the AD type patients had the highest incidence of aMCI (90.9%, *n* = 10/11) and aMCI multiple domain ratio (81.8%, *n* = 9/11) while hippocampal volumes were the smallest. The number of patients who progressed to dementia was the greatest in the follow-up period (90.9%, *n* = 10/11). An explanation for this is that at baseline, cognitive function in this group was the poorest, so the possibility that more patients with late stages of MCI was included in this group is likely, but it is also likely that the patients in this group are rapidly progressing to dementia. This is because the median onset age for this group is 65 years, so not only could there be a large number of patients in the prodromal stage of early-onset Alzheimer's disease (EOAD), but evidence from several studies indicates that patients with EOAD have a potentially more aggressive clinical course (Stanley and Walker, 2014). Many of the patients of the AD type are likely to progress to the hippocampal sparing type (Murray et al., 2011) and parietal dominant type (Noh et al., 2014) identified by previous cluster analysis studies, and patients in these types are known to progress more rapidly (Murray et al., 2011; Na et al., 2016).

Neurodegenerative disorders are due to pathologic processes related to abnormal protein accumulation and activation of aberrant biochemical, metabolic and structural changes. Role of neuroinflammation has emerged in the development of neurodegenerative disorders (Cerami et al., 2017). Most widely studied neuroinflammation PET is translocator protein (TSPO) imaging, which has been known to detect the activated microglia. In the majority of the studies, an increase in TSPO binding was observed in AD dementia vs. age-matched healthy controls and was correlated with cognitive functions or cerebral gray matter (Janssen and Mach, 2019). However, in case of MCI patients, results were inconsistent and controversial. In some studies, MCI patients showed elevated TSPO binding compared to healthy controls, however, in other studies, they failed to earn significant differences from healthy controls (Schain and Kreisl, 2017). It is thought to reflect the TSPO PET signal may increase or decrease between initial peak and late peak in the neuroinflammation process in the disease progression. Otherwise, subtypes of microglial cells, i.e., M1 which plays a proinflammatory role, and M2 which plays an anti-inflammatory role, may have an effect (Calsolaro and Edison, 2016). Contrary to TSPO imaging, [^18^F]THK5351 reflects combination of tau and astrogliosis and shows more robust results showing elevated retention in the clinically corresponding brain regions and changes according to disease severity (Kang et al., 2017; Huang et al., 2019; Park et al., 2019; Jeong et al., 2020).

We acknowledge several limitations to the findings of this study. First, diagnosis based on pathologic confirmations was absent and the patients with amyloid PET data represented just 40.6% of the study subjects. Second, the small number of study subjects limits generalizability of this study. Further studies with a larger sample size and pathologic data would be needed to draw confirmative conclusions. Finally, as all the study subjects did not undertook follow-up detailed neuropsychological tests, we could not quantitate the disease progression in each group.

In conclusion, it appears possible to identify clinically distinctive subgroups of MCI patients and those patients who are at greater risk of dementia progression using cluster analyses of [^18^F]THK5351 retention patterns. [^18^F]THK5351 PET imaging which assesses both astrogliosis and tau may be applicable to discriminate neurodegenerative diseases in MCI status.

## Data Availability Statement

The original contributions presented in the study are included in the article/Supplementary Material, further inquiries can be directed to the corresponding author, Prof. Young Noh.

## Ethics Statement

The studies involving human participants were reviewed and approved by Gachon University Gil Medical Center, IRB Committee. The patients/participants provided their written informed consent to participate in this study.

## Author Contributions

YN, J-KS, and DN: conceptualization. W-RK, H-ES, and S-YL: data curation. HL, E-CL, SS, and K-PK: formal analysis. YN: funding acquisition. J-KS and K-PK: methodology. YN and J-KS: resources. K-PK, J-KS, and NO: validation. HL and E-CL: writing-original draft. YN, J-KS, and NO: writing-review and editing. All authors contributed to the article and approved the submitted version.

## Conflict of Interest

The authors declare that the research was conducted in the absence of any commercial or financial relationships that could be construed as a potential conflict of interest.

## Abbreviations

MCI: mild cognitive impairment
AD: Alzheimer's disease
NC: normal cognition
aMCI: amnestic mild cognitive impairment
OR: odds ratio
NFT: neurofibrillary tangle
MAO-B: monoamine oxidase B
FLUTE: flutemetamol
SUVR: standard uptake value ratio
ROI: region-of-interest
MMSE: mini-mental state examination
CDR SOB: clinical dementia rating–sum of boxes
CTh: cortical thickness
WMH: white matter hyperintensity
DWMH: deep white matter hyperintensity
PWMH: periventricular white matter hyperintensity.
