# Supplementary material for: Identification of Heterogeneous Subtypes of Mild Cognitive Impairment Using Cluster Analyses Based on PET Imaging of Tau and Astrogliosis
Source: Front Aging Neurosci. 2021 Jan 26;12:615467. doi: 10.3389/fnagi.2020.615467 (PMC7874013; doi:10.3389/fnagi.2020.615467)
Supplement: Supplementary file 1 [file Data_Sheet_1.pdf]

Supplementary Material

Data S1. Neuropsychological tests

Neuropsychological testing was performed using the Seoul Neuropsychological Screening Battery (SNSB)-II (Kang et al., 2012). This battery contains assessments of attention (forward and backward Digit Span Test [DST]), language function (Korean-Boston Naming Test [K-BNT]), praxis(Ideomotor, Buccofacial), the four symptoms testing of Gerstmann syndrome (Writing, Finger naming, Right-left orientation and calculation), visuospatial function (Rey Complex Figure Test[RCFT]-copy), verbal memory (Seoul Verbal Learning Test-Elderly's version[SVLT-E] immediate, 20-min delayed recall and recognition), visual memory([RCFT] immediate, 20-min delayed recall and recognition), and frontal/executive functions (semantic and phonemic Controlled Oral Word Association Test [COWAT]; Korean-Color Word Stroop Test[K-CWST]; Korean-Trail Making Test-Elderly's version[K-TMT-E]) (Ahn et al., 2010).

Data S2. Assessment of lacunes and microbleeds on MRI

Lacunes were defined as lesions of ≥3 mm and ≤15 mm in diameter with low signal on T1-weighted images, high signal on T2-weighted images, and a perilesional halo on 80 axial sections of FLAIR images. Microbleeds were defined as small lesions of ≤10 mm in diameter, using criteria proposed by Greenberg *et al.*(Greenberg et al., 2009) on 20 axial slices of T2* gradient-recall echo MR images. The numbers of lacunes and microbleeds were counted in four lobar white matter (frontal, parietal, temporal, and occipital), thalamus, basal ganglia and infratentorial regions. Lacunes or microbleeds in the thalamus and basal ganglia were incorporated into the frontal region because the thalamus and basal ganglia are part of the fronto-subcortical circuit.

Reference

Ahn, H.J., Chin, J., Park, A., Lee, B.H., Suh, M.K., Seo, S.W., et al. (2010). Seoul Neuropsychological Screening Battery-dementia version (SNSB-D): a useful tool for assessing and monitoring cognitive impairments in dementia patients. J Korean Med Sci 25(7), 1071-1076. doi: 10.3346/jkms.2010.25.7.1071.

Greenberg, S.M., Vernooij, M.W., Cordonnier, C., Viswanathan, A., Salman, R.A.-S., Warach, S., et al. (2009). Cerebral microbleeds: a guide to detection and interpretation. *The Lancet Neurology* 8(2), 165-174.

Kang, Y., Jahng, S., and Na, D.L. (2012). *In: Seoul Neuropsychological Screening Battery, 2nd Edition (SNSB-II).* Seoul: Human Brain Research & Consulting Co.
